# An Iron Transporter Is Involved in Iron Homeostasis, Energy Metabolism, Oxidative Stress, and Metacyclogenesis in *Trypanosoma cruzi*

**DOI:** 10.3389/fcimb.2021.789401

**Published:** 2022-01-10

**Authors:** Claudia F. Dick, Nathália Rocco-Machado, André L. A. Dos-Santos, Luiz F. Carvalho-Kelly, Carolina L. Alcantara, Narcisa L. Cunha-E-Silva, José R. Meyer-Fernandes, Adalberto Vieyra

**Affiliations:** ^1^ Leopoldo de Meis Institute of Medical Biochemistry, Federal University of Rio de Janeiro, Rio de Janeiro, Brazil; ^2^ Carlos Chagas Filho Institute of Biophysics, Federal University of Rio de Janeiro, Rio de Janeiro, Brazil; ^3^ National Center of Structural Biology and Bioimaging (CENABIO), Federal University of Rio de Janeiro, Rio de Janeiro, Brazil; ^4^ Graduate Program in Translational Biomedicine/BIOTRANS, Unigranrio University, Duque de Caxias, Brazil

**Keywords:** iron transporter, *Trypanosoma cruzi*, reactive O_2_ species production, parasite O_2_ consumption, parasite proliferation, parasite differentiation, maintenance of *T. cruzi* infection

## Abstract

The parasite *Trypanosoma cruzi* causes Chagas’ disease; both heme and ionic Fe are required for its optimal growth, differentiation, and invasion. Fe is an essential cofactor in many metabolic pathways. Fe is also harmful due to catalyzing the formation of reactive O_2_ species; for this reason, all living systems develop mechanisms to control the uptake, metabolism, and storage of Fe. However, there is limited information available on Fe uptake by *T. cruzi*. Here, we identified a putative 39-kDa Fe transporter *in T. cruzi* genome, TcIT, homologous to the Fe transporter in *Leishmania amazonensis* and *Arabidopsis thaliana*. Epimastigotes grown in Fe-depleted medium have increased *TcIT* transcription compared with controls grown in regular medium. Intracellular Fe concentration in cells maintained in Fe-depleted medium is lower than in controls, and there is a lower O_2_ consumption. Epimastigotes overexpressing TcIT, which was encountered in the parasite plasma membrane, have high intracellular Fe content, high O_2_ consumption—especially in phosphorylating conditions, high intracellular ATP, very high H_2_O_2_ production, and stimulated transition to trypomastigotes. The investigation of the mechanisms of Fe transport at the cellular and molecular levels will assist in elucidating Fe metabolism in *T. cruzi* and the involvement of its transport in the differentiation from epimastigotes to trypomastigotes, virulence, and maintenance/progression of the infection.

## 1 Introduction

*Trypanosoma cruzi*, the etiological agent of Chagas’ disease, has a complex life cycle, alternating between an intermediate invertebrate host and a definitive mammalian host (de Souza, 1984). *Trypanosoma cruzi* has a high requirement for iron (Fe) for proliferation, *in vitro* and *in vivo*, mobilizing heme and non-heme iron (Lalonde and Holbein, 1984). *Trypanosoma cruzi* can hijack Fe-proteins from a mammalian host. The addition of deferoxamine, an Fe chelator, or transferrin-free serum can inhibit the proliferation of amastigote cells in culture, an indication that Fe is an obligatory nutrient (Lima and Villalta, 1990). This form of the parasite presents receptors for human transferrin, which bind exogenous transferrin. Transferrin bound to amastigote cells is not removed by acid treatment, indicating a possible internalization and utilization of this transferrin (Lima and Villalta, 1990). In epimastigotes, transferrin uptake occurs through the cytostome, a specialized structure composed of a membrane invagination in the anterior region, close to the flagellar pocket (Porto-Carreiro et al., 2000), an early observation showing that Fe-carrying molecules are important for the parasite in its different morphological stages and, therefore, for the establishment, maintenance, and evolution of the infection in the vertebrate host. Heme is also utilized as an Fe source for *T. cruzi*; it can stimulate *T. cruzi* proliferation in culture in a dose-dependent manner (Lara et al., 2007). Moreover, heme/porphyrin translocates in epimastigote forms of *T. cruzi*, possibly mediated by an ABC transporter protein (Cupello et al., 2011). However, the limiting step for the utilization of heme by pathogenic trypanosomatids is the initial heminic ring hydrolysis for Fe liberation (Taylor and Kelly, 2010), since there is no heme oxidase gene in the *T. cruzi* genome (El-Sayed et al., 2005).

Before direct Fe incorporation by cells, Fe^3+^ (the predominant redox form in nature) has to be reduced to Fe^2+^, catalyzed by an Fe-reductase (Sedlácek et al., 2009). In this way, the identification of an Fe-reductase activity in *Leishmania chagasi* (Wilson et al., 2002), *Leishmania amazonensis* (Flannery et al., 2011), and more recently, *T. cruzi* (Dick et al., 2020) is strongly indicative of a Fe^2+^ transport mechanism in trypanosomatids. Due to the Fe low redox potential (*E*° = −0.04 V for the reaction Fe^3+^ + 3e^−^ → Fe), it becomes a suitable element for redox catalysis reactions (Atkins and de Paula, 2006), where it can act as an electron donor and receptor. The ability to pass readily through oxidation/reduction cycles leads to the inherent toxicity of Fe, since it catalyzes the formation of reactive O_2_ species (ROS) as hydroxyl radical (OH^•^), which provides a high redox potential (*E*° = +2.33 V for the reaction OH^•^ + e^−^ + H^+^ → H_2_O) (Krumova and Cosa, 2016), *via* the Fenton reaction.

Regarding Fe metabolism in *T. cruzi*, there is strong evidence that it is important in the catalysis by antioxidant defenses (Paiva et al., 2012). Superoxide dismutase isoforms (SODs) are metalloproteins that can dismutate ${\text{O}}_{2}^{\cdot -}$ in H_2_O_2_ and O_2_. Metallic ions are present in the catalytic center from SODs, e.g., Cu-Zn-SOD in eukaryotes (Tainer et al., 1983) and Mn-SOD in bacteria, such as *Escherichia coli* (Geslin et al., 2001), whereas trypanosomatids exclusively have Fe-SOD, with Fe as the cofactor (Wilkinson et al., 2006). However, there is little information regarding Fe uptake in *T. cruzi* (Sutak et al., 2008) and its role in i) the differentiation process that is required for the infection of the vertebrate host and ii) the stimulus of oxidative stress within the parasite, which favors this differentiation process.

We have identified a sequence putatively annotated as the Zn/Fe transporter. *In-silico* analysis demonstrated its homology to the Fe transporters described in *L. amazonensis* (Huynh et al., 2006) and *Arabidopsis thaliana* (Vert et al., 2002). It is the first identification of an Fe transporter (TcIT) homologous to LIT1 possibly responsible for Fe uptake in *T. cruzi*, working coupled to the Fe-reductase TcFR recently described in our laboratory (Dick et al., 2020). We hypothesize that this transporter may be important in the maintenance and progression of *T. cruzi* infection.

## 2 Materials and Methods

### Epimastigote Growth and Metacyclogenesis

Epimastigotes of *T. cruzi* (Dm28c strain) were maintained at 28°C in stationary phase by using brain heart infusion (BHI) medium supplemented with 10% FBS, 30 μM hemin, and 1% penicillin–streptomycin cocktail (regular medium, RM). Iron-depleted medium (IDM) was prepared using BHI medium without hemin and supplemented with 10% iron-free FBS, following the protocol described in Dick et al. (2020). The viability of the parasites was assayed by evaluating the mitochondrial transmembrane potential. When this potential is generated and maintained, the 3-(4,5-dimethylthiazol)-2,5-diphenyltetrazolium (MTT) bromide (Sigma-Aldrich, Saint Louis, MO, USA) is converted to insoluble formazan. The concentration of formazan was spectrophotometrically determined at 570 nm after its solubilization with Triton X-100.

For the assay of epimastigote proliferation, the parasites were inoculated (10^6^ cells/ml) on the 6th day of culture into the BHI medium (RM or IDM, with or without G418). Cell proliferation was assessed every day by counting the number of cells in a hemocytometer. Metacyclogenesis was induced as described in Contreras et al. (1985) and Koeller et al. (2014). Briefly, epimastigotes in transition from logarithmic to stationary phase were adjusted to 5 × 10^8^ parasites/ml in triatomine artificial urine (TAU) medium [190 mM NaCl, 17 mM KCl, 2 mM MgCl_2_, 2 mM CaCl_2_, 0.035% (w/v) NaHCO_3_, and 8 mM phosphate buffer at pH 6.0]. After 2 h at 28°C, the cultures were diluted 100-fold in 10 ml TAU medium supplemented with 10 mM L-proline, 50 mM glutamic acid, 2 mM aspartic acid and 10 mM glucose (TAU3AAG), and 500 µg/ml G418 (Sigma-Aldrich) and transferred to T25 flasks—lying at an angle of 45° to increase the area in contact with O_2_—and maintained at 28°C to promote metacyclogenesis. After 3–5 days, the parasites were quantified by hemocytometry, and the percentage of metacyclic trypomastigotes was estimated by their morphology after Giemsa staining.

The percentage of trypomastigotes was also quantified by cytometry as previously described (Bayer-Santos et al., 2013). Briefly, live parasites (4 × 10^7^) were incubated for 30 min on ice with the monoclonal antibody 1G7 against GP90, diluted in 1% bovine serum albumin in phosphate-buffered saline (BSA/PBS). Later, the cells were washed in PBS and were fixed with 4% paraformaldehyde (PFA) in PBS for 15 min. Then, after washings in PBS, the parasites were incubated with Alexa Fluor 488-conjugated anti-mouse IgG diluted in 1% BSA/PBS for 1 h at room temperature. Subsequently, after two more washes, fluorescence was determined on a FACSCalibur II cytometer (Becton Dickinson, Franklin Lakes, NJ, USA), and data analysis was performed using the CellQuest software (Becton Dickinson).

### *In-Silico* Analysis

From the *in-silico* analysis of the genome of the Dm28c strain of *T. cruzi* (available through the TriTryp database under the accession no. TCDM_06386) (Grisard et al., 2014), we found a homolog (TcIT) to the Fe transporter of *L. major* (available from the TriTryp database under the accession no. LmjF.31.3070-LIT). The model of TcIT was constructed using the protein structure prediction PHYRE (www.sbg.bio.ic.ac.uk/phyre/) (Kelley and Sternberg, 2009), which is based on the model of the SERCA ATPase and visualized with the standard molecular viewer PyMOL 2002 (PyMOL Molecular Graphics System, DeLano Scientific, San Carlos, CA, USA; http://pymol.sourceforge.net/). Phylogenetic analysis used the MEGA 7 software. The evolutionary history was inferred using the neighbor-joining method (Saitou and Nei, 1987). Amino-acid multiple sequence alignments were obtained by using Clustal W and Clustal X software version 2.0 (http://www.ebi.ac.uk/Tools/msa/clustalw2).

### Cloning and Overexpression of TcIT in *Trypanosoma cruzi*

The full-length TcIT coding region was amplified from Dm28c gDNA, by using designated primers FTcIT (5′-GGATCCATGAACAACGTTGAGTCAAGTGACGCG-CACCT) introducing the *Bam*HI restriction site at the 5′-end and RTcITHA (5′-AAGCTTTTAAGCGTAATCTGGAACATCGTATGGGTACGCCCACTTCCCAAGGAGCGTCATAA), with the addition of *Hin*dIII restriction site with the hemagglutinin (HA) epitope tag at the 3′-end, thus generating the TcIT-HA insert. The amplicon was subcloned into pCR2.1-TOPO vector (Thermo Fisher Scientific, Waltham, MA, USA), released by digestion with *Bam*HI and *Hin*dIII (sites underlined above), and ligated into similarly digested expression vector pTEX (Kelly et al., 1992). The shuttle vector pTEX-TcIT-HA, which replicates in *E. coli* and *T. cruzi*, was used as the vehicle for the expression of TcIT-HA in *T. cruzi*. Cell electroporation was performed with an Amaxa Nucleofector II device with human T-cell buffer (Lonza, Basel, Switzerland). A total of 5 × 10^7^ epimastigotes were transfected with pTEX-TcIT-HA or empty pTEX (pTEX-Ø) vectors (10 μg DNA). After electroporation, the cells were cultured for 48 h in standard medium and 500 µg/ml G418 (Sigma-Aldrich) was added. Non-DNA control cells died after 3 to 4 weeks. Cultures were 5-fold diluted with fresh G418-containing medium after 5–10 days. Stable resistant cells were obtained ~30 days after transfection, indicating resistance to G418.

### Intracellular Iron Concentration Determination

The concentration of intracellular Fe accumulated under different conditions and by different strains (wild type and mutants obtained as described above) was determined by a colorimetric assay based on the use of ferrozine. Suspensions containing 10^8^ parasites were collected from different cultures and washed three times with PBS pretreated with 5 g/100 ml Chelex resin (Sigma-Aldrich). The cells were lysed with 100 μl 50 mM NaOH, followed by the addition of 100 μl 10 mM HCl; the release of ionic Fe bound to intracellular structures was achieved by adding 100 μl of a mixture of 1.4 M HCl and 4.5% (w/v) KMnO_4_ (1:1) to the cell lysate, followed by incubation at 60°C for 2 h. Then, 30 μl Fe detection reagent (6.5 mM ferrozine, 6.5 mM neocuproine, 2.5 M ammonium acetate, and 1 M ascorbic acid) was added. After 30 min of incubation at room temperature, the absorbance of the sample was recorded at 550 nm. The concentration of Fe was determined using a standard curve with known FeCl_3_ concentrations (0–75 μM) (Mittra et al., 2013).

### Membrane Fraction Preparation

Mutant epimastigotes (5 × 10^9^ cells) in late log phase of growth were harvested by centrifugation and washed three times in cold PBS. The plasma membrane fraction/PM was obtained as previously reported (Gottlieb and Dwyer, 1983; Pinheiro et al., 2006; Dick et al., 2020), with slight modifications. The washed organisms, overexpressing pTEX-TcIT or pTEX-Ø, were resuspended in Tris–EDTA buffer (10 mM Tris–HCl at pH 8.0, 125 mM sucrose, 3 mM MgCl_2_, 2 mM EDTA, and 1 mM phenylmethanesulfonyl fluoride) and maintained on ice for 30 min. The parasites were mixed with glass beads (1:4) and disrupted by abrasion for 10 min on an ice bath. After grinding, glass beads, unbroken cells, and large cell debris were removed by centrifugation at 1,000×*g* for 15 min at 4°C. The supernatant (total homogenate/HG) was centrifuged at 200,000×*g* for 1 h. The resulting pellet (total membranes/TM) was resuspended in 50 mM Tris–HCl (pH 8.0) and subsequently applied to a continuous density gradient of 18% Percoll in 0.25 M sucrose and 12 mM Tris–HCl (pH 7.4), to obtain the PM-enriched fraction. After centrifugation at 40,000×*g* for 1 h, the bands were removed by aspiration, analyzed for 5′-nucleotidase activity [the marker of PM in epimastigote forms of *T. cruzi* (Zingales et al., 1979) and Fe-reductase activity, and the two fractions (TM and PM) were kept at −80°C, along with aliquots of HG, for further assays. Protein concentration was determined by the Lowry method (Lowry et al., 1951), using BSA as standard. **Supplementary Figure S1** depicts the assays of 5′-nucleotidase and Fe-reductase activities, demonstrating i) the enrichment of the PM fraction with both enzymes and ii) that these activities were barely detectable in the supernatant recovered after centrifugation of HG at 200,000×*g* for 1 h (the cytosolic fraction/C).

### Western Blotting and Immunolocalization

For Western blotting detection, the proteins of the fractions obtained as described above (30 µg/lane) were separated by 12% SDS-PAGE and transferred to nitrocellulose membranes (Merck Millipore, Burlington, MA, USA), which were blocked with 5% milk in PBS plus 0.1% (w/v) Tween 20, probed overnight at 4°C with the primary rabbit anti-HA antibody (1:1,000, Sigma-Aldrich), and detected using an HRP-conjugated anti-rabbit IgG secondary antibody (1:10,000, Santa Cruz Biotechnology, Dallas, TX, USA). Ponceau red was used as loading control.

Immunofluorescence was assayed as previously described, with minor modifications (Dick et al., 2020). The mutant *T. cruzi* epimastigotes (10^7^ cells, pTEX-TcIT or pTEX-Ø) were washed three times in 0.5 ml PBS and the suspension was fixed with 500 μl formaldehyde 4% (w/v) in PBS for 1 h at room temperature. The samples were washed twice with 0.5 ml PBS, suspended in 40 μl PBS, and settled on poly-L-lysine-coated coverslips for 15 min. The coverslips were incubated in 0.4% (w/v) saponin in PGN [PBS at pH 7.2, supplemented with 0.2% (w/v) gelatin and 0.1% (w/v) NaN_3_] for 15 min to allow parasite permeabilization. The samples were incubated with anti-HA antibody raised in rabbit (1:1,000, Sigma-Aldrich) and mouse anti-TcSMP (surface membrane proteins) antibody (1:100), prepared and used as described by Martins et al. (2015) in PGN with 0.1% saponin, and incubated overnight at 4°C. After washing with PGN, the samples were incubated with anti-rabbit FITC conjugated (1:100, Sigma-Aldrich) and anti-mouse Alexa 594 (1:1,000, Sigma-Aldrich) in PGN for 1 h at room temperature. Coverslips were washed in PBS and incubated with 0.1 µg/ml 4′,6-diamino-2-phenylindole (DAPI, Sigma-Aldrich) for 30 min. After washing, coverslips were mounted on slides in Miowol (Antifade) reagent. Images were taken with a Leica TCS-SPE confocal microscope and processed with Leica confocal software.

### Transmission Electron Microscopy

Cells were fixed by using 2.5% (v/v) glutaraldehyde in 0.1 M cacodylate buffer (pH 7.2) for 1 h at room temperature and post-fixed using an osmium-thiocarbohydrazide-osmium (OTO) protocol (Willingham and Rutherford, 1984). Briefly, the cells were incubated in a post-fixative solution [1% (v/v) OsO_4_, 0.8% (v/v) potassium ferrocyanide, and 5 mM CaCl_2_ in 0.1 M cacodylate buffer (pH 7.2)] for 40 min, washed twice in water, and then incubated in a solution of 1% (w/v) thiocarbohydrazide (TCH, Sigma-Aldrich) in water for 5 min. After three washes in water, the cells were incubated again in the post-fixative solution for 3 min. Samples were dehydrated in an acetone series and embedded in epoxy resin. Ultrathin sections (70 nm) were stained post-embedding with 5% (w/v) uranyl acetate and lead citrate and observed by using a Tecnai Spirit electron microscope (FEI Co., Hillsboro, OR, USA) operating at 120 kV.

### Real-Time PCR

Total *T. cruzi* RNA was extracted using a Direct-zol RNA MiniPrep Kit (Zymo Research, Orange, CA, USA) from epimastigotes maintained at RM or IDM for 6 days, or mutants pTEX-TcIT or pTEX-Ø (as indicated in the figure legends). Total RNA was subjected to reverse transcription using the High-Capacity cDNA reverse transcription kit (Thermo Fisher Scientific, Waltham, MA, USA). For RT-PCR, 100 ng/μl cDNA per well was used (15 μl total volume), along with 5 μM primer mix and 7 μl PowerUp SYBR green master mix (Thermo Fisher Scientific). The primers 5′-TCTGGTCGC-TTCTCTTCTCG and 5′-TAAAGA-CTCCGGCACACAGT were used to amplify a 152-bp fragment of the *TcIT* gene. The primers 5′-AGCGCGCGTCTAAGACTTACA and 5′-TG-GAGCTGCGGTTGTCATT that amplify the glyceraldehyde-3-phosphate dehydrogenase (GAPDH) constitutive gene were used to provide an endogenous control.

### High-Resolution Respirometry in Different Respiratory States

Oxygen consumption was measured using intact epimastigotes (5 × 10^7^ parasites/chamber; pTEX-TcIT or pTEX-Ø). Analyses used an O2k-system high-resolution oxygraph (Oroboros Instruments, Innsbruck, Austria). The cells were suspended in 2 ml respiration solution containing 100 mM sucrose, 50 mM KCl, and 50 mM Tris–HCl (pH 7.2) at 28°C with continuous stirring, and 50 μM digitonin was added to permeabilize the parasites. Oxygen concentrations and O_2_ consumption were recorded using DatLab software coupled to Oxygraph-2K (basal O_2_ consumption). Subsequently, 10 mM succinate and 200 μM ADP were added. Uncoupled respiration was stimulated after adding 3 μM FCCP and respiration was inhibited by adding 2.5 μg/ml antimycin A to measure residual O_2_ consumption (Nogueira et al., 2017). In a series of experiments, titration with substrates and inhibitors was not carried out, and O_2_ consumption was measured only in the presence of endogenous substrates (basal consumption) over all recordings.

### Superoxide Dismutase Activity

Superoxide dismutase (SOD) activity was measured as described in Winterbourn et al. (1975), with modifications, based on SOD inhibiting the reduction of nitro blue tetrazolium (NBT) by ${\text{O}}_{2}^{\cdot -}$ . Epimastigote cells were harvested by centrifugation, washed three times in cold PBS, and disrupted by freeze–thaw. The protein concentration of the total homogenate was quantified by the Lowry method (Lowry et al., 1951). The homogenates (using known quantities of protein in the range of 10–50 µg) were incubated in reaction medium (200 µl, final volume) containing 45 mM potassium phosphate buffer (pH 7.8), 6.5 mM EDTA, and 50 mM NBT. The reaction was initiated by adding 2 mM riboflavin. After 12 min in a light box, the absorbance of the sample was recorded at 560 nm. The percent inhibition was measured for each amount of protein, and SOD activity was expressed as the amount of enzyme inhibiting NBT reduction by 50%.

### Intracellular ATP Quantification

The intracellular ATP was quantified by using an ATP bioluminescent somatic cell assay kit (Sigma-Aldrich). Briefly, mutant epimastigotes (10^7^ parasites per tube, 0.1 ml) were incubated in a solution containing 100 mM sucrose, 50 mM KCl, and 50 mM Tris–HCl (pH 7.2 adjusted with HCl). Cellular extracts were prepared by mixing 0.1 ml epimastigotes with 0.1 ml somatic cell ATP releasing reagent and the mixture was left on ice for 1 min. Half of the cellular extract (0.1 ml) was transferred to MTS-11C minitubes (Axygen, Union City, CA, USA) containing 0.1 ml ATP assay mix and stirred for 10 s at room temperature. The total amount of light emitted was measured with a GloMax Multi JR detection system (Promega, Madison, WI, USA). Total intracellular ATP concentration per cell number was calculated using a standard ATP curve, prepared, and analyzed in each experiment (Carvalho-Kelly et al., 2020).

### Amplex Red Peroxidase Assay

The production of H_2_O_2_ in mutants overexpressing TcIT was assayed by the rate of H_2_O_2_ reduction to H_2_O, which is stoichiometrically coupled (1:1) to the simultaneous oxidation of the non-fluorescent Amplex^®^ Red probe to the fluorescent resorufin (Dos-Santos et al., 2019). Briefly, assays containing 0.1 μM H_2_O_2_ were incubated with 10^7^ parasites/ml for 30 min at room temperature in 5 mM Tris–HCl (pH 7.4), 1.7 μM Amplex^®^ Red (Invitrogen, Carlsbad, CA, USA), and 6.7 U/ml horseradish peroxidase (Sigma-Aldrich) in a final volume of 100 μl. The evolution of fluorescence was followed at excitation/emission wavelengths of 563/587 nm (slit 5 nm).

### Interaction of Trypomastigotes With LLC-MK2 Cells

Metacyclogenesis was induced as described above in section 2.1. After 3 days, the parasites were collected and purified using the ion-exchange chromatography technique Sepharose membrane-DEAE (Cruz-Saavedra et al., 2017) to obtain a preparation enriched with metacyclic trypomastigotes. Then, the parasites were incubated with LLC-MK2 cells (CCL-7; ATCC, Rockville, MD, USA) (50 parasites:1 cell) in RPMI 1640 medium supplemented with 10% FBS in a 96-well PS F-bottom microplate (Greiner Bio-One Brazil Ltd., Americana, Brazil) for 24 h at 37°C under 5% CO_2_ in air. After removing the medium containing parasites not adhered to the cells, adding the fresh medium, and incubating further (48 h), the cells were fixed with 4% paraformaldehyde in PBS for 10 min. After incubation with Hoechst 33342 (Invitrogen) (1:5,000 dilution) for 60 min at room temperature in the dark, the wells were washed with Milli-Q deionized water and analyzed in a high-content screening system ImageXpress Micro XL (Molecular Devices, San José, CA, USA) using the MetaXpress 6.0 software. The interaction parasites:cells was quantified by counting the respective nuclei.

### Statistical Analysis

Data are presented as mean ± SE. Means were compared by using unpaired Student’s *t*-test and GraphPad Prism 7.0 (San Diego, CA, USA). When more than two means were compared, one-way ANOVA followed by Tukey’s test or two-way ANOVA with multiple comparisons was used, as indicated in the text or in the figure legends.

## 3 Results

### Identification and Analysis of Iron Transporter Sequence in *Trypanosoma cruzi*

**Figure 1:**
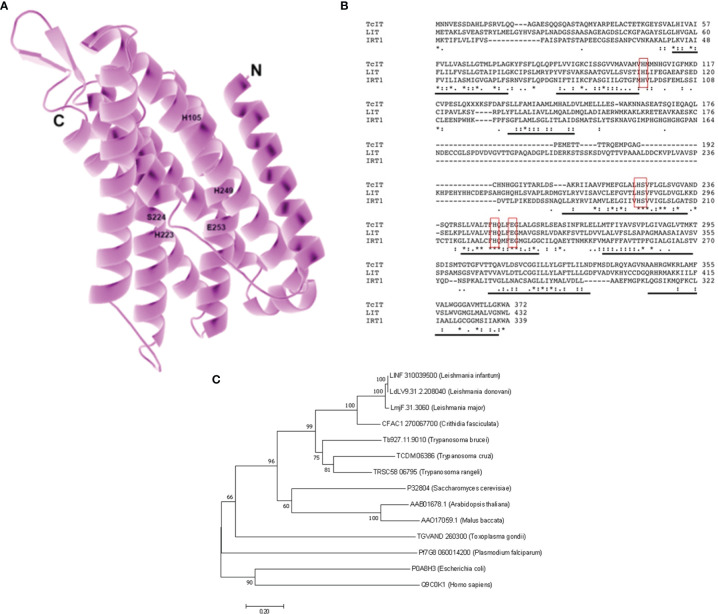
Model for the iron (Fe) transporter TcIT: alignment and phylogenetic analysis for TcIT and ZIP family members from different species. **(A)** Structural model of Fe transporter from *T. cruzi* (TcIT). The model was constructed using the protein structure prediction PHYRE (www.sbg.bio.ic.ac.uk/phyre/) (Kelley and Sternberg, 2009) and visualized with the standard molecular viewer PyMOL 2002 (PyMOL Molecular Graphics System, DeLano Scientific, San Carlos, CA, USA; http://pymol.sourceforge.net/). The eight transmembrane helices are shown in pink, and labels highlight the five key residues mentioned in the text. **(B)** Alignment of amino-acid sequences from *T. cruzi* TcIT, DM28c strain (TriTryDB: TCDM_06386), *L. major* LIT1 (TritrypDB: LmjF.31.3060), and *Arabidopsis thaliana* IRT1 (GenBank: AAB01678.1). Conserved residues responsible for Fe transport in IRT1 and LIT1 (Jacques et al., 2010) are indicated in red boxes. Predicted transmembrane domains are underlined in black. Alignment used the ClustalW algorithm. Identical amino acids are indicated with an asterisk (*). Conservation between amino acid groups of strongly similar properties is indicated by a colon (:). Conservation between amino acid groups of weakly similar properties is indicated by a period (.). **(C)** Evolutionary relationships of ZIP family members. The evolutionary history was inferred using the neighbor-joining method (Saitou and Nei, 1987). The optimal tree with the sum of branch length = 6.09506879 is shown. The percentage of replicate trees in which the associated taxa clustered together in the bootstrap test (1,000 replicates) is shown at the beginning of each branch (Felsenstein, 1985). The tree is drawn to scale, with branch lengths in the same units as those of the evolutionary distances used to infer the phylogenetic tree. The evolutionary distances were computed using the Poisson correction method and given in the units of the number of amino-acid substitutions per site. All positions containing gaps and missing data have been eliminated. Evolutionary analyses were conducted in MEGA7 (Kumar et al., 2016), which involved 14 amino-acid sequences: *Leishmania infantum* putative sequence (GenBank: LINF310039500), *L. donovani* putative sequence (GenBank: LdLV9.31.2.208040), *L. major* LIT1 (GenBank: LmjF.31.3060), *Crithidia fasciculata* putative sequence (GenBank: CFAC1 270067700), *Trypanosoma brucei* putative sequence (GenBank: Tb927.11.9010), *T. cruzi* TcIT (GenBank: TCDM06386, this work), *T. rangeli* putative sequence (GenBank: TRSC58 06795), *Saccharomyces cerevisiae* ZRT1 (GenBank: P32804), *Arabidopsis thaliana* IRT1 (GenBank: AAB01678.1), *Malus baccata* MbIRT1 (GenBank: AAO17059.1), *Toxoplasma gondii* putative sequence (GenBank: TGVAND 260300), *Plasmodium falciparum* (GenBank: PfG8 0600 14200), *Escherichia coli* ZupT (GenBank: P0A8H3), and *Homo sapiens* SLC39A8 HsZIP8 (GenBank: Q9C0K1).

A putative zinc–iron (Zn–Fe) transporter sequence was found in the genome database of T. cruzi (TriTrypDB: TCDM_06386) following a BLAST search, using the Fe transporter LIT1 from L. amazonensis (TritrypDB: LmjF.31.3060) (Huynh et al., 2006) as target. The TCDM_06386 was named TcIT and has 1,119 bp, and the deduced amino-acid sequence of the peptide comprises 372 residues, thus resulting in a predicted molecular mass of 39.8 kDa. The deduced protein TcIT has a structural model (**Figure 1A**) in which its eight possible transmembrane domains can be seen (TMpred Server) (Hofmann and Stoffel, 1993). TcIT has a highly conserved domain corresponding to the zinc iron permeases (ZIP) superfamily domain (permeases classified as 2.A.5 in the Transporter Classification Database/TCDB, Saier Lab. Bioinformatics Group). We encountered the domain in Zn and Fe transporter proteins by using the Conserved Domain Database (CDD, National Center for Biotechnology Information/NCBI). This domain has conserved amino acids corresponding to Zn/Fe binding, such as those labeled H105, H223, S224, H249, and E253 in **Figure 1A**, which are located along the inner surface of the channel formed by the transmembrane domains and bind the metal during the transport process (Jacques et al., 2010).

TcIT alignment had 34% identity and 53% similarity to the LIT1 from L. amazonensis and 28% identity and 46% similarity to the IRT1 from A. thaliana (**Figure 1B**), with 30% identity between the sequences of L. amazonensis and A. thaliana (Jacques et al., 2010). Analysis showed that all three sequences have eight transmembrane domains (underlined in **Figure 1B**) and the same residues for Zn/Fe binding (residues H105, H223, S224, H249, and E253, red boxes in **Figure 1B**). To analyze the phylogenetic relationship with the ZIP superfamily, we compared the amino-acid sequence of TcIT with sequences of putative and functionally characterized Fe transporters from several organisms, using MEGA 7 software (**Figure 1C**).

### Low Iron Availability Induces the Expression of TcIT

**Figure 2:**
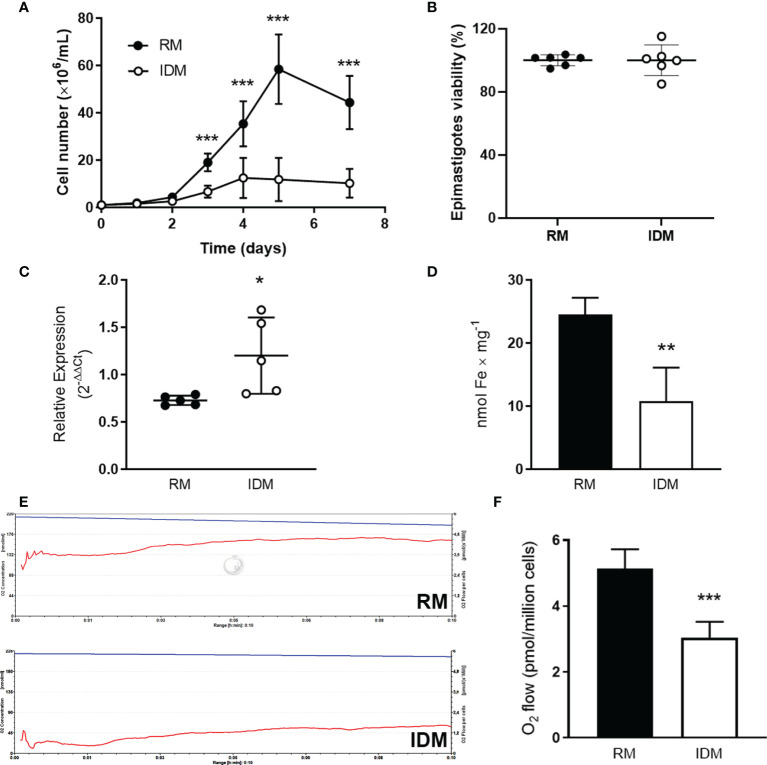
Growth and respiration of wild-type epimastigotes are dependent on Fe content in culture medium and Fe depletion increases the expression of TcIT. **(A)** Influence of culture medium Fe content on *T. cruzi* proliferation. Wild-type epimastigotes of *T. cruzi* at mid-log phase were harvested, washed twice, seeded in fresh medium, and grown for the indicated times in regular medium [RM: brain heart infusion medium (BHI) supplemented with 30 µM hemin and 10% fetal bovine serum (FBS)] (filled circles) or in iron-depleted medium (IDM: BHI without hemin supplementation and treated with Chelex for ionic Fe depletion plus 10% iron-depleted FBS) (empty circles); ***P < 0.001 (*n* = 12). **(B)** Viability of epimastigotes incubated in RM (filled circles) or IDM (empty circles) (5 × 10^7^ epimastigotes/ml) was assayed using the MTT assay. **(C)** Quantification of the TcIT transcript in wild-type *T. cruzi* epimastigotes. Quantitative PCR was done using 100 ng cDNA from epimastigotes in mid-log phase when maintained in RM (filled circles) or IDM (empty circles); *P < 0.05 (*n* = 5). The housekeeping *GADPH* gene was used to normalize qPCR. **(D)** Intracellular Fe content in epimastigotes maintained in RM (black bars) or IDM (empty bars); **P < 0.01 (*n* = 6). **(E)** Representative recordings of O_2_ consumption rates vs. time (pmol/s per cell; lower traces in both panels) and decrease of O_2_ concentration (nmol/ml; upper traces in both panels): 5 × 10^7^ epimastigotes/chamber from RM (upper panel) or IDM (lower panel) were harvested, washed twice, and used to measure O_2_ consumption in reaction medium (2 ml) containing 116 mM NaCl, 5.4 mM KCl, 5.5 mM D-glucose, 50 mM HEPES–Tris (pH 7.2). **(F)** Quantification of O_2_ consumption by epimastigotes incubated in RM (black bars) or IDM (empty bars) (***P < 0.001; *n* = 6). In all cases, using unpaired Student’s *t*-test assessed differences between mean values. In **(A)**, the differences were assessed by comparing time-matched determinations.

Impaired growth was seen in epimastigotes maintained in IDM compared with those grown in RM (**Figure 2A**). The decrease in cell number was not due to cell death: MTT assays showed that viability of the cells was similar (**Figure 2B**). Although cells in IDM possess TcIT transcripts that increased with Fe removal from the culture medium (**Figure 2C**), this was insufficient for maintaining Fe intracellular content: epimastigotes from IDM had 50% lower intracellular Fe content compared with epimastigotes from RM (**Figure 2D**). In addition, Fe depletion lowered basal O_2_ consumption (representative recordings in **Figure 2E**), comparatively quantified in **Figure 2F**.

### Plasma Membrane Localization of TcIT

**Figure 3:**
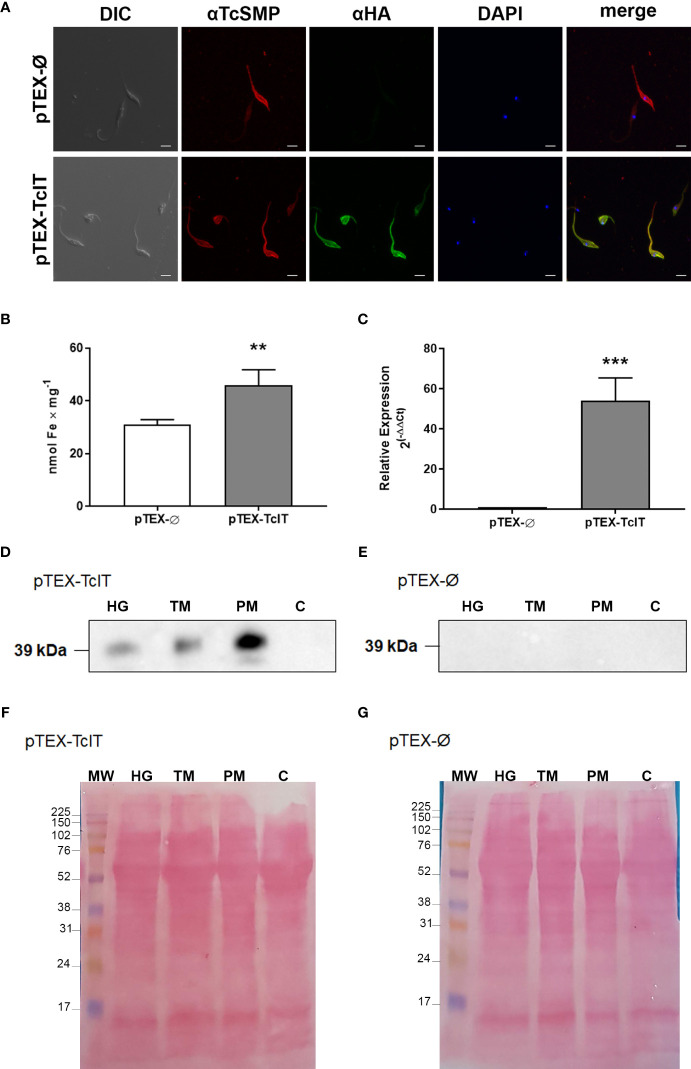
**(A)** Immunofluorescence of epimastigotes shows TcIT expression in plasma membranes. *Trypanosoma cruzi* epimastigotes were transfected with empty vector (pTEX-Ø) or TcIT tagged with hemagglutinin (pTEX-TcIT). DIC: differential interference contrast microscopy. TcSMP: images using antisera for this surface membrane protein and the secondary anti-mouse-Alexa 546 (red). HA: TcIT expression using anti-HA and secondary anti-rabbit FITC (green). DAPI: nuclei stained with DAPI (blue). In merge, the green fluorescent staining for TcIT overlaps with the red fluorescent signal for TcSMP, suggesting the proteins are in the same region of the plasma membrane; ×60 magnification, scale bar 4 μm. **(B)** Intracellular Fe content is higher in epimastigotes transfected with pTEX-Ø (empty bar) or pTEX-TcIT (gray bar); **P < 0.01 (*n* = 6). **(C)** Quantification of the TcIT transcript in *T. cruzi* epimastigotes. Quantitative PCR was carried out using 100 ng cDNA from mutants pTEX-Ø (empty bar) or pTEX-TcIT (gray bar); ***P < 0.001 (*n* = 4). In both cases, using Student’s *t*-test assessed differences between mean values. **(D)** Representative Western blot using homogenate (HG), total membrane (TM), plasma membrane (PM), and cytosol **(C)** fractions from pTEX-TcIT epimastigotes, as indicated in the upper part of the image, and the antibody anti-HA. **(E)** Representative Western blot using HG, TM, PM, and C fractions from pTEX-Ø epimastigotes, as indicated in the upper part of the image, and the antibody anti-HA. The predicted size of TcIT is 39.8 kDa. **(F, G)** Ponceau red loading controls for representative Western blots of membranes from pTEX-TcIT and pTEX-Ø epimastigotes, as indicated in the upper left corners.

By using immunofluorescence assays, we determined that the labeling of the tagged protein TcIT-HA colocalizes with the labeling of the plasma membrane protein TcSMP, indicating that the Fe transporter is localized in the plasma membrane of epimastigotes (**Figure 3A**).

As negative control, parasites transfected with pTEX-Ø did not stain with HA. Mutants overexpressing TcIT had a higher Fe intracellular content compared with the basal content of the mutant transfected with pTEX-Ø (**Figure 3B**), together with very high transcription levels of TcIT (**Figure 3C**). In agreement with the immunofluorescence results, the plasma membrane-enriched fraction (PM) from pTEX-TcIT had a 39-kDa band corresponding to TcIT. **Figure 3D** shows a representative Western blot image, where the arrow points to the 39-kDa band; no bands were observed in pTEX-Ø epimastigotes (**Figure 3E**). The densitometric analysis of pTEX-TcIT Western blots (*n* = 3) of the HG, total membranes, and PM gave, respectively, the following values in arbitrary units: 52.9 ± 8.6, 59.6 ± 2.4, and 133.0 ± 2.9. One-way ANOVA followed by Tukey*’*s test demonstrated that the enrichment of TcIT in PM with respect to HG (160%) and TM (130%) was highly significant (*P* < 0.0001 and *P* = 0.0001, respectively). Ponceau red loading controls are presented in **Figures 3F** (pTEX-TcIT) and **Figure 3G** (pTEX-Ø).

### TcIT Transporter Increases Mitochondrial Respiratory Rates in *Trypanosoma cruzi*

**Figure 4:**
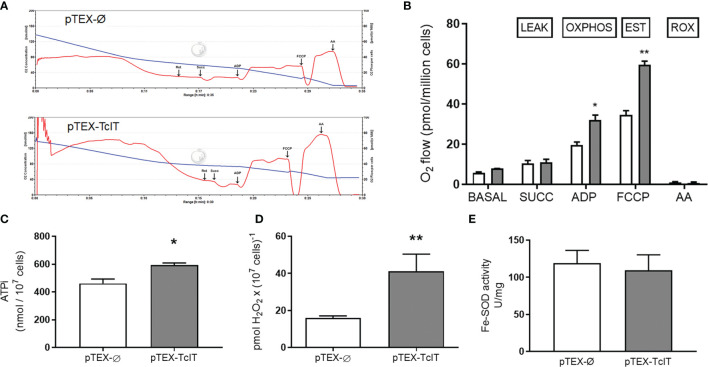
Effect of TcIT overexpression on epimastigote mitochondrial physiology and Fe-SOD activity. **(A)** Representative recordings of O_2_ consumption and decrease of O_2_ concentration. *Trypanosoma cruzi* epimastigotes transfected with pTEX-Ø empty vector (upper panel) or pTEX-TcIT plasmid (lower panel) were initially tested for respiration of intact epimastigotes. Epimastigotes were then digitonin-permeabilized, as described in Nogueira et al. (2017), to measure mitochondrial activity after the successive additions indicated by arrows: Rot (rotenone), Succ (succinate), ADP, FCCP, and AA (antimycin A). **(B)** Graphic representation of O_2_ consumption after the additions shown on the abscissa: SUCC, ADP, FCCP, and AA; **P < 0.01, *P < 0.05 (*n* = 4). **(C)** Intracellular ATP in *T. cruzi* epimastigotes transfected with pTEX-Ø empty vector (empty bar) or pTEX-TcIT plasmid (gray bar) was measured as described in *M&M* section. *P < 0.05 (*n* = 3). **(D)** Production of H_2_O_2_ by living cells of *T. cruzi* epimastigotes transfected with pTEX-Ø empty vector (empty bar) or pTEX-TcIT plasmid (gray bar); **P < 0.001 (*n* = 3). **(E)** SOD activity was assessed from epimastigotes transfected with pTEX-Ø empty vector (empty bars) or pTEX-TcIT plasmid (gray bars). The values represent the mean ± SEM. In **(C–E)**, using Student’s *t*-test assessed differences between mean values. In **(B)**, differences were assessed by two-way ANOVA followed by Tukey’s test. In all cases, the uncoupled respiration (with FCCP) was significantly higher than the respiration in phosphorylating conditions (with ADP). Both stimulated respiratory states were also significantly higher than those obtained only after addition of succinate (at least *P* < 0.05). No differences were found between the respiration of pTEX-Ø pTEX-TcIT epimastigotes in the presence of endogenous substrates or after the addition of succinate.

In **Figures 2E, F**, we showed that Fe depletion in culture decreases O_2_ consumption by T. cruzi. **Figures 4A** (representative traces) and **Figure 4B** (quantification of O_2_ consumption in different respiratory states) show that higher respiration rates are encountered—in phosphorylating and uncoupled conditions—when TcIT is overexpressed. Permeabilization with digitonin did not alter the profile of basal respiration (data not shown). After adding succinate (the LEAK O_2_ consumption), there was no difference in O_2_ consumption between pTEX-Ø and pTEX-TcIT epimastigotes, but the subsequent addition of ADP (corresponding to the oxidative phosphorylation—OXPHOS—state) that caused a significant increase of respiration by the two classes of mutants, O_2_ consumption of TcIT mutants was higher compared with pTEX-Ø mutants. There were a further increase and uncoupled respiration rates (EST, electron transfer system uncoupled from phosphorylation) after the addition of FCCP (a H^+^ ionophore), again higher in overexpressing TcIT. The addition of antimycin A completely abolished mitochondrial respiration in both mutants.

Higher mitochondrial O_2_ consumption can be coupled to both increased oxidative phosphorylation and higher ROS production. Therefore, we measured total intracellular ATP concentration (**Figure 4C**) and H2O2 formation (**Figure 4D**) in both mutants. Parasites overexpressing TcIT had higher intracellular ATP (ATPi) content, as was expected, and a 2-fold higher content of H_2_O_2_. Nevertheless, there was no difference between the mutants in their activity of the antioxidant enzyme SOD (**Figure 4E**).

**Figure 5:**
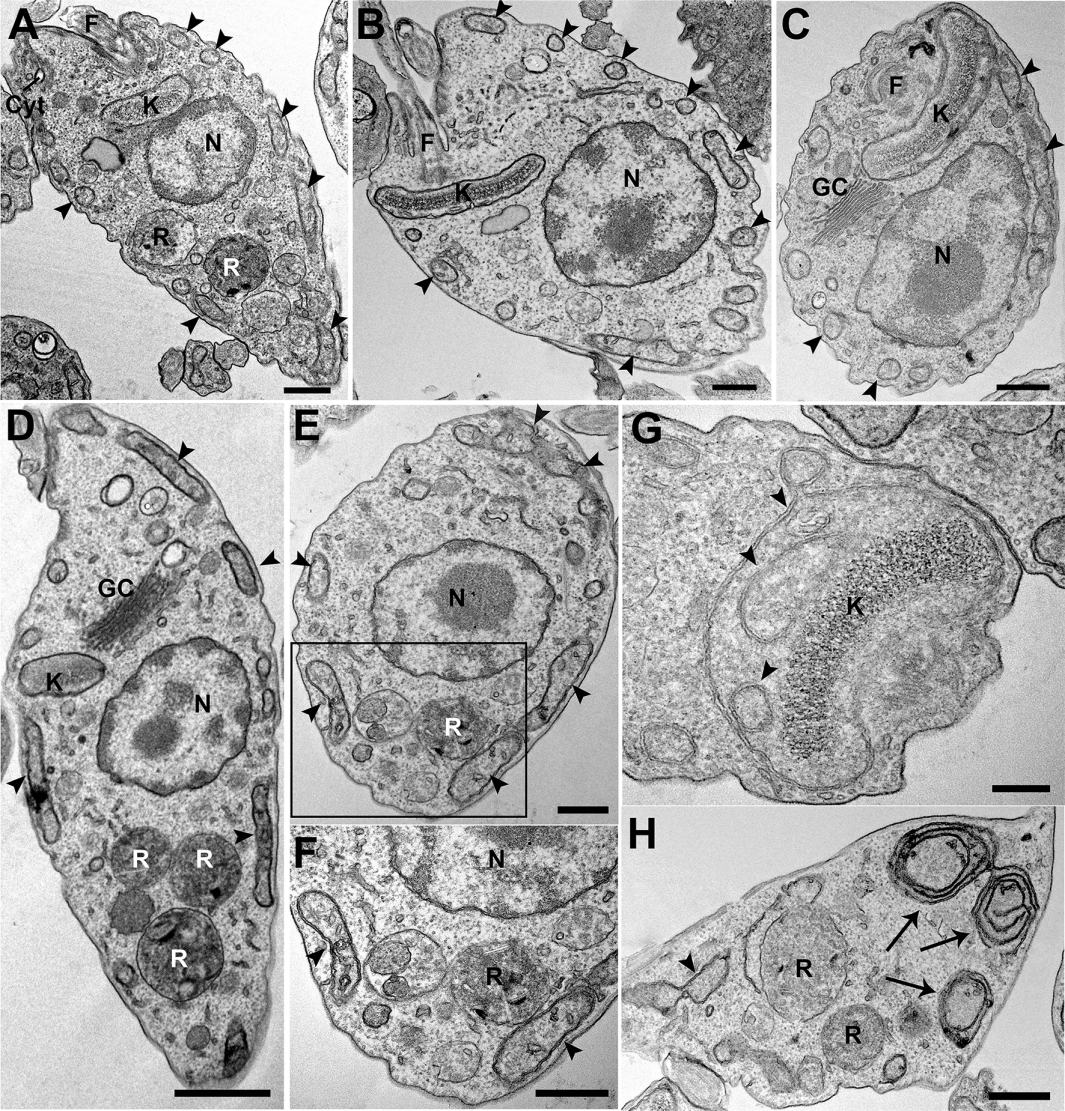
Ultrastructural changes in TcIT-superexpressing parasites. pTEX-Ø and epimastigotes overexpressing TcIT were processed and observed by transmission electron microscopy. **(A–C)** Control pTEX-Ø epimastigotes show normal morphology of major cellular organelles: kinetoplast (K), nucleus (N), Golgi complex (GC), cytostome–cytopharynx complex (Cyt), and reservosomes (R). Mitochondrial branches are seen all along the cell body, juxtaposing the cell membrane (arrowheads). F (flagellum). **(D–H)** Epimastigotes overexpressing TcIT. **(D)** Mutants also present normal organelle morphology and positioning when compared with controls, except for the mitochondria. **(E)** Swollen mitochondrial branches (arrowheads) are observed in overexpressing parasites. **(F)** Increased magnification of the area delimited by the rectangle in **(E)** shows an increase in mitochondrial cristae and inner membrane vesiculation (arrowheads). **(G)** Mitochondrial inner vesiculation was observed at the kinetoplast (K) region (arrowheads). **(H)** Areas of mitochondrial inner membrane disorganization were also observed (arrows). Bars: **(A–C, E, F, H)** 500 nm; **(D)** 1 µm; **(G)** 200 nm.

Possible ultrastructural changes in epimastigotes that overexpress TcIT were analyzed by transmission electron microscopy (**Figure 5**). As control, we used parasites transfected with pTEX-Ø. Control epimastigotes presented normal cell and organelle morphology and positioning (**Figures 5A–C**). We could not find any alteration in the major cellular organelles, including kinetoplast, nucleus, and organelles of the endocytic pathway such as cytostome–cytopharynx complex and reservosomes. Most organelles had normal morphology and positioning in epimastigotes that overexpress TcIT (**Figures 5D–H**), except for the mitochondria. In mutant parasites, we frequently observed mitochondrial swelling, increase in cristae density, inner membrane vesiculation (**Figures 5E–G**), and mitochondrial disorganization (**Figure 5H**). These findings may reflect the increase in mitochondrial activity found in the case of parasites overexpressing TcIT.

### Identification of the *Trypanosoma cruzi* Epimastigote Phenotype Overexpressing the TcIT Transporter: Stimulus of Differentiation to Trypomastigote and Replication Amastigote Rate

**Figure 6:**
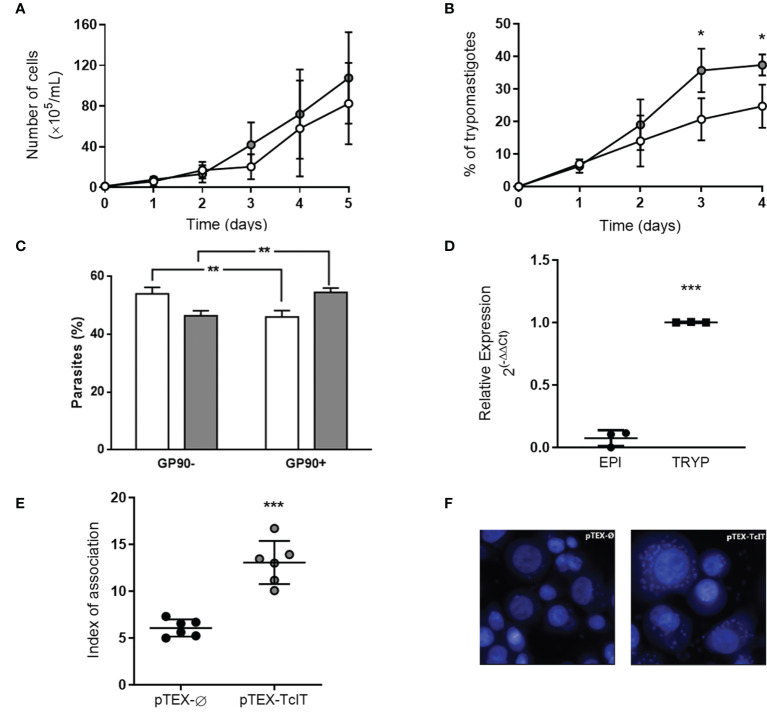
TcIT overexpression influences *T. cruzi* differentiation to trypomastigotes, but not proliferation. **(A)** *T. cruzi* epimastigotes transfected with pTEX-Ø empty vector (white circles) or pTEX-TcIT plasmid (gray circles) proliferation. Epimastigotes of *T. cruzi* at mid-log phase were harvested, washed twice, seeded into fresh medium, and grown for the indicated times in regular medium under the antibiotic pressure of G418 (50 mg/ml) (*n* = 6). **(B)** Transfected epimastigotes with empty vector pTEX-Ø (white circles) or pTEX-TcIT (gray circles) were exposed to *in-vitro* differentiation medium TAU. Each day, epimastigotes and trypomastigotes were differentially counted to determine the percentage of trypomastigotes in culture; *P < 0.05 (*n* = 4). **(C)** Cytometric analysis of parasites after the 4th day in TAU **(B)** using the 1G7 antibody against the marker of metacyclogenesis GP90. **(D)** Quantification of the TcIT transcript in *T. cruzi* epimastigotes (EPI) or trypomastigotes (TRYP). Quantitative PCR used 100 ng cDNA from mutants, as indicated on the abscissa; ***P < 0.001 (*n* = 3). **(E)** Index of association estimated by total number of intracellular parasites per infected LLC-MK2 cells. ***P < 0.001 (*n* = 6). **(F)** Representative images of pTEX-Ø or pTEX-TcIT parasites infection in LLC-MK2 cells, quantitatively compared in **(E)**. Except for **(C)**, using Student’s *t*-test assessed the differences between mean values. In **(A, B)**, the differences were assessed by comparing time-matched determinations. In **(C)**, the comparisons were performed by using two-way ANOVA. **P < 0.01 (*n* = 3).

Overexpression of TcIT in T. cruzi epimastigotes did not interfere with their in-vitro proliferation (**Figure 6A**), since these mutants proliferate at the same rate as the epimastigotes transfected with the empty vector pTEX-Ø. However, when epimastigotes transfected with pTEX-TcIT were subjected to in-vitro differentiation in TAU medium, almost 40% of the forms present after 96 h were trypomastigotes, whereas transfection with pTEX-Ø for the same time led to only 20% of the organisms as trypomastigotes (**Figure 6B**). This indicates that overexpression of the TcIT transporter favors in-vitro differentiation. The cytometric analysis (**Figure 6C**) using the 1G7 antibody against the marker of metacyclogenesis GP90 demonstrated, after the 4*th* day in TAU, a higher number of GP90(+) cells in parasites transfected with TcIT than in those transfected with pTEX-Ø (54.5% vs. 46%). In contrast, the number of GP90(−) cells was higher in pTEX-Ø parasites (54% vs. 46.4%). The hypothesis that TcIT overexpression stimulates metacyclogenesis is also supported by the fact that trypomastigotes have higher levels of TcIT mRNA than epimastigote forms (**Figure 6D**). In fact, LLC-MK2 cells infected with metacyclic trypomastigotes derived from epimastigotes pTEX-*TcIT present a* higher replication rate of amastigotes (parasites/LLC-MK2 cells) when compared with those LLC-MK2 cells infected with pTEX-Ø *metacyclics* (**Figure 6E**). *Representative* images of the amastigote forms inside LLC-MK2 cells are shown in **Figure 6F**.

## 4 Discussion

*Trypanosoma cruzi* has a high requirement for Fe, mobilizing heme or non-heme Fe, for both *in-vitro* proliferation of epimastigotes and *in-vivo* proliferation in mice (Lalonde and Holbein, 1984). In mammalian cells, Fe is complexed with proteins, e.g., ferritin, which has the highest number of bound Fe per molecule of protein; hemoglobin, which carries the largest amount of Fe in the body; lactoferrin, mainly found in mucosae; and finally, transferrin (Tf), a seric protein responsible for transporting Fe to all cells (Weinberg, 2009). The identification of a Tf receptor in *T. cruzi* amastigote forms (Lima and Villalta, 1990) and the fact that these forms can take up human transferrin directly by the endocytosis pathway *via* the cytostome (Porto-Carreiro et al., 2000; Rocha et al., 2010) indicate that *T. cruzi* amastigotes can use Tf as the major Fe source. *Trypanosoma cruzi* may take up hemin through an ABC transporter (Lara et al., 2007; Cupello et al., 2011). Before heme can be utilized, its degradation by heme oxidase (HO) is necessary; however, the *HO* gene is absent in the *T. cruzi* genome (El-Sayed et al., 2005), limiting Fe removal from the heme ring (Taylor and Kelly, 2010). In this way, reducing Fe^3+^ to Fe^2+^ releases the ion from the extracellular hemin, thus facilitating Fe uptake by heavy-metal transporters, as proposed for *Leishmania* parasites (Ortíz-Estrada et al., 2012). Identifying Fe-reductase TcFR in *T. cruzi* plasma membranes (Dick et al., 2020) opened up the possibility of the same mechanism being present in the parasite.

Ferric ion reduction to Fe^2+^ by Fe-reductases is usually coupled to Fe^2+^ transport in bacteria, yeasts, plants, and animal cells (Huynh and Andrews, 2008). The identification of an Fe-reductase in *L. chagasi* (Wilson et al., 2002), *L. amazonensis* (Flannery et al., 2011), and recently, in *T. cruzi* (Dick et al., 2020) strongly suggests that trypanosomatids have Fe^2+^ transport systems. In this way, the identification of a putative Fe transporter in the *T. cruzi* genome, TcIT (TriTryDB: TCDM_06386) modeled in **Figure 1A**, which is homologous to the newly described Fe transporter in *L. amazonensis*, LIT (TritrypDB: LmjF.31.3060), and the *Arabidopsis thaliana* Fe transporter IRT1 (GenBank: AAB01678.1), supports this idea. Indeed, analysis of the predicted amino-acid sequence shows eight transmembrane domains, as shown for TcIT, LIT, and IRT1 sequences (**Figure 1B**). Both *Leishmania* LIT1 and *Arabidopsis* IRT1 are Fe^2+^ transporters from the ZIP family, and the amino- and carboxyl-terminal ends are located on the extracellular side of the plasma membrane. By alignment analysis, we found that, as in LIT1 and IRT1, *T. cruzi* TcIT has the same features. The most conserved portion of ZIP family proteins is also present; the amphipathic helix of the putative transmembrane domain IV contains a His residue and an adjacent semipolar Ser residue, essential components for heavy-metal binding sites (Guerinot, 2000; Huynh et al., 2006). Thus, TcIT is the first member of the ZIP family to be identified in *T. cruzi*.

A wide range of biological processes, such as electron and O_2_ transport and DNA synthesis, depend on Fe content, making Fe homeostasis an ensemble of processes that are essential for all cells, including pathogenic protozoa (Nadadur et al., 2008; Ortíz-Estrada et al., 2012). Upregulation of TcIT mRNA levels in response to Fe depletion (**Figure 2C**) indicated that the parasite has a compensatory mechanism, although insufficient for sustaining the Fe requirement (**Figure 2D**), possibly because the Fe content is very low in IDM. Low O_2_ consumption in parasites maintained in Fe-depleted medium (**Figure 2F**) suggests that Fe depletion arrests mitochondrial function. Plasma membrane immunolocalization (**Figure 3A**) and enrichment in the plasma-membrane fraction (**Figure 3D**) also strongly suggest that TcIT is responsible for Fe uptake in *T. cruzi* epimastigotes, ensuring differentiation to infective trypomastigotes. TcIT could be translocated to the inner mitochondrial membrane *via* an unknown mechanism involving membrane fusion despite the evident immunolocalization in the parasite plasma membrane. In addition, Fe^2+^ could reach the mitochondria through an unknown iron transporter present in the inner mitochondrial membrane or bound to a carrier protein or metalloprotein. This could be one of the ways to allow Fe to interact with the mitochondrial complexes, thus stimulating respiration and H_2_O_2_ formation.

Parasites overexpressing TcIT (**Figure 6D**) have a higher capacity for differentiating into trypomastigotes *in vitro* (**Figures 6B, C**). These forms express proteins (such as gp83 glycoprotein, cruzipain, oligopeptidase B, and P21) that are involved with host-cell invasion (Burleigh and Andrews, 1998; Yoshida and Cortez, 2008; da Silva et al., 2009; Martins et al., 2020). In this regard, and as an example of maintenance of chronic infection, it is of interest that P21 was recently demonstrated to be important for intracellular confinement of *T. cruzi* amastigotes (Martins et al., 2020). In addition, parasites that overexpress TcIT display an increase in the average number of intracellular parasites per LLC-MK2-infected cells after 48 h post-infection (**Figures 6E, F**), demonstrating the importance of Fe transporter in increasing the rate of amastigote replication.

In *Leishmania*, upregulation of LIT1 expression activates promastigote to amastigote differentiation, mainly *via* Fe-SOD activity. Fe-SOD activity converts ${\text{O}}_{2}^{\cdot -}$ to H_2_O_2_, a molecule that triggers the generation of infective amastigotes (Mittra et al., 2013). Although H_2_O_2_ production is very high in parasites that overexpress TcIT (**Figure 4D**), Fe-SOD activity is not modulated by the presence of the gene (**Figure 4E**), indicating that elevated levels of H_2_O_2_ are the result of accelerated dismutation of an elevated ${\text{O}}_{2}^{\cdot -}$ formation. In *T cruzi*, the significant increase in H_2_O_2_ production seems to be due to mitochondrial activity (**Figures 4A, B**). Indeed, the mitochondria were the only organelles where significant changes were observed (**Figure 5**). We propose that the 200% increase in H_2_O_2_ could be key for *T. cruzi* infectivity and virulence, as demonstrated by the persistence of amastigotes in macrophages of infected mice (Paiva et al., 2012). Since heme induces epimastigote proliferation *via* mitochondrial ROS production without altering ATP production (Nogueira et al., 2017), it may be that TcIT overexpression induces epimastigote to trypomastigote differentiation through a different signaling pathway. Therefore, we conclude that this work presents evidence regarding the possible participation of TcIT in Fe metabolism, proliferation/differentiation processes, infectivity virulence, and maintenance of *T. cruzi* infection. Since the lack of correlation between the increase of TcIT expression in IDM and the Fe content in the parasite raises the question of whether the protein is the only mechanism responsible for Fe uptake in *T. cruzi*, further transport or generation and analysis of mutant cell lines need to be performed in the future.

**Figure 7:**
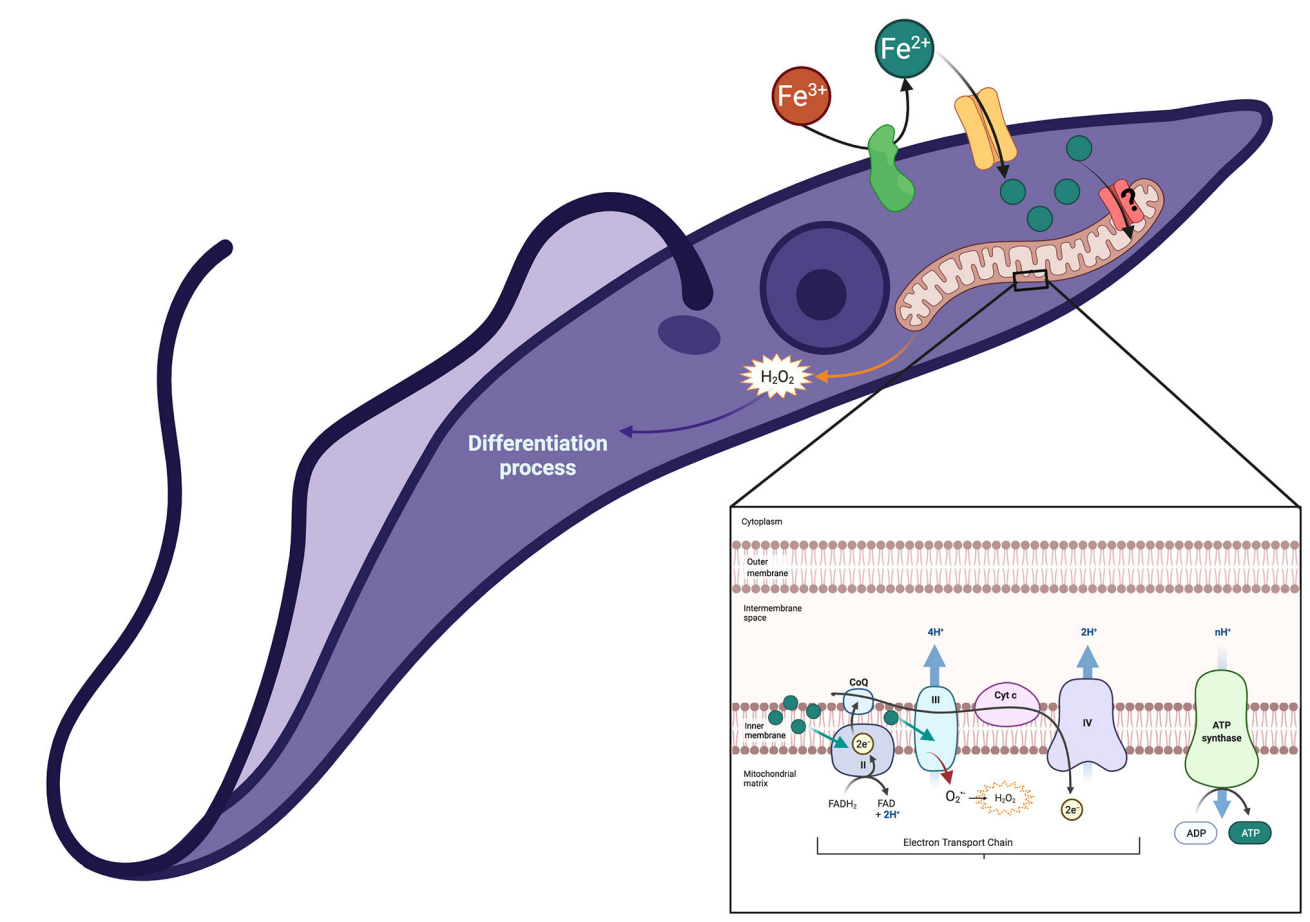
Proposed model of functional coupling between TcFR and TcIT in the plasma membrane of *T. cruzi*, which allows Fe^3+^ reduction, Fe^2+^ uptake, its delivery to the cytosol, and through reaching the mitochondria either *via* an unknown iron transporter present in the inner mitochondrial membrane or bound to a carrier protein or metalloprotein. Once in the mitochondria, Fe^2+^ stimulates H_2_O_2_ formation from an excess of the substrate ${\text{O}}_{2}^{\cdot -}$ , and differentiation to trypomastigote forms, as depicted in the amplified graphical insert of a mitochondrial structure. Green filled circles represent Fe^2+^ ions entering the mitochondrial matrix *via* internalized TcIT molecules that could insert in the mitochondrial internal membrane after their cycling into the cytosol. H_2_O_2_-induced mitochondrial remodeling could be responsible for the activation of differentiation, as recently proposed for other scenarios where epimastigotes differentiate into trypomastigotes (Pedra-Rezende et al., 2021). Created with BioRender.com.

Finally, **Figure 7** presents a hypothetical mechanistic model for the functional coupling between TcFR (Dick et al., 2020) and TcIT for Fe^2+^ uptake across the plasma membrane of *T. cruzi*, which increases mitochondrial H_2_O_2_ formation and stimulates parasite differentiation, thus allowing invasion of the host cells.

## Data Availability Statement

The original contributions presented in the study are included in the article/**Supplementary Material**. Further inquiries can be directed to the corresponding author.

## Author Contributions

CD conceived, designed, and performed the experiments; analyzed the data; and wrote the manuscript. NR-M, AD-S, LC-K, and CA contributed to the experimental process. CA and NC-S analyzed the data and revised the manuscript. JM-F and AV conceived the experiments, analyzed the data, and revised the manuscript. All authors contributed to the article and approved the submitted version.

## Funding

This research was funded by the Brazilian National Research Council/CNPq to CD (grant Young Talented People # 300338/2015-5), JM-F (grant # 401134/2014-8), and AV (grant # 307605/2015-9). The support of the Rio de Janeiro State Research Foundation/FAPERJ to CD (grant Grade 10 for Post-doctoral Studies # E-26/202.359/2017), JM-F (grant # E-26/201.300/2014), and AV (grant # E-26/2012.963/2017) is also acknowledged.

## Conflict of Interest

The authors declare that the research was conducted in the absence of any commercial or financial relationships that could be construed as a potential conflict of interest.

## Publisher’s Note

All claims expressed in this article are solely those of the authors and do not necessarily represent those of their affiliated organizations, or those of the publisher, the editors and the reviewers. Any product that may be evaluated in this article, or claim that may be made by its manufacturer, is not guaranteed or endorsed by the publisher.

## Supplementary Material

Supplementary Figure 1 **(A)** 5’-nucleotidase activity in fractions of pTEX-Ø (n = 4). **(B)** 5’-nucleotidase activity in fractions of pTEX-TcIT (n = 4). **(C)** Fe-reductase activity in fractions of pTEX-Ø (n = 3). **(D)** Fe-reductase activity in fractions of pTEX-TcIT (n = 3). HG, total homogenate; TM, total membranes; PM, plasma membranes; C, cytosol. In all cases, differences were assayed by one-way ANOVA following by Tukey’s test within each panel. NS, not significant; **P < 0.01; ***P < 0.001.
