# Supplementary figures and images for: An Iron Transporter Is Involved in Iron Homeostasis, Energy Metabolism, Oxidative Stress, and Metacyclogenesis in Trypanosoma cruzi
Source: Front Cell Infect Microbiol. 2022 Jan 10;11:789401. doi: 10.3389/fcimb.2021.789401 (PMC8785980; doi:10.3389/fcimb.2021.789401)

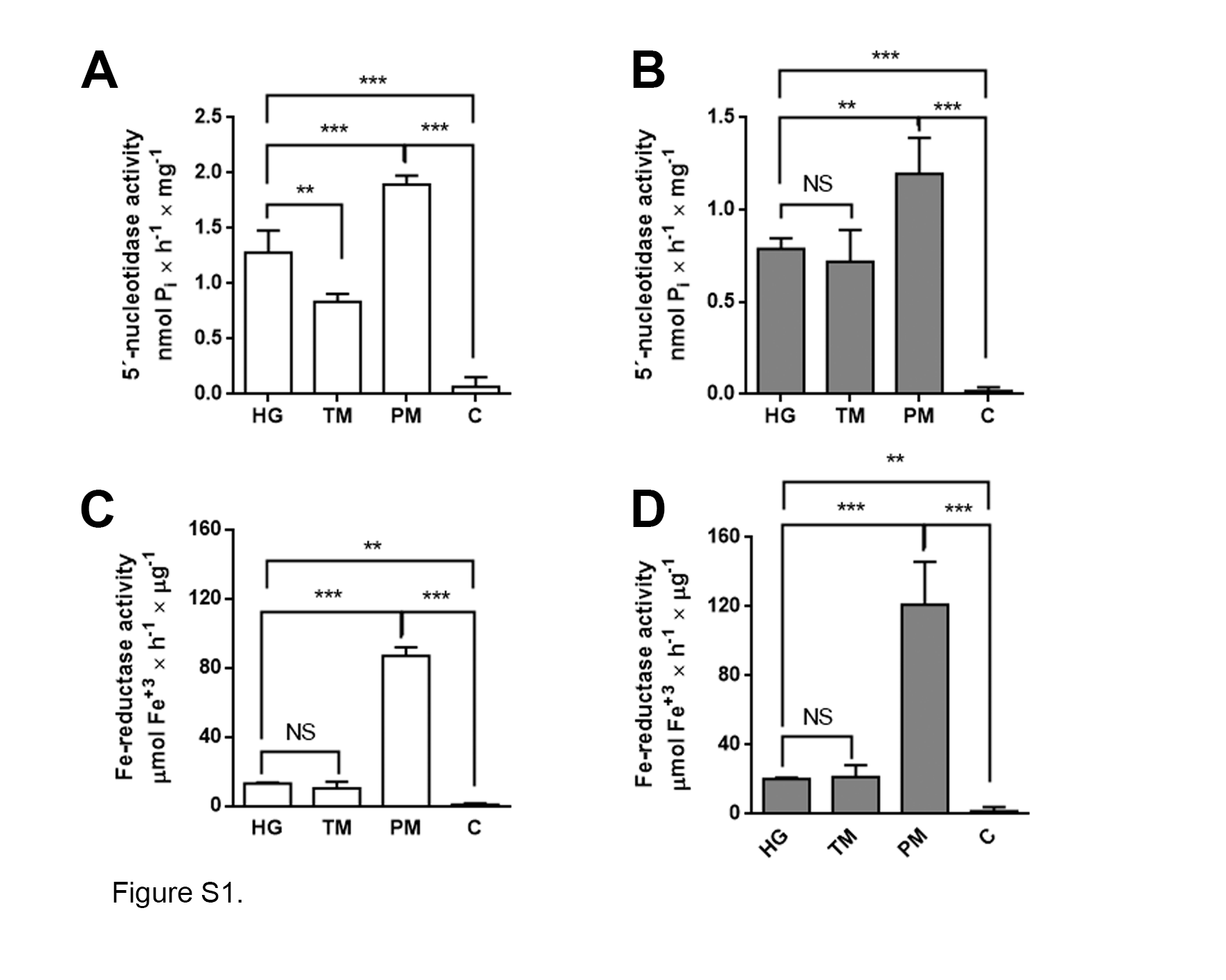

Supplement: Supplementary Figure 1 — (A) 5’-nucleotidase activity in fractions of pTEX-Ø (n = 4). (B) 5’-nucleotidase activity in fractions of pTEX-TcIT (n = 4). (C) Fe-reductase activity in fractions of pTEX-Ø (n = 3). (D) Fe-reductase activity in fractions of pTEX-TcIT (n = 3). HG, total homogenate; TM, total membranes; PM, plasma membranes; C, cytosol. In all cases, differences were assayed by one-way ANOVA following by Tukey’s test within each panel. NS, not significant; **P < 0.01; ***P < 0.001. [file Image_1.tif]
